# Supplementary material for: Apoptotic neurodegeneration in whitefly promotes the spread of TYLCV
Source: eLife. 2020 Jul 30;9:e56168. doi: 10.7554/eLife.56168 (PMC7392610; doi:10.7554/eLife.56168)
Supplement: Supplementary file 1. [file elife-56168-supp1.docx]

**Supplementary file 1.** Oligonucleotides list

| Name | Primer Sequence (5’-3’)^a^ | Purpose and No. |
| --- | --- | --- |
| V61 | ATACTTGGACACCTAATGGC | For DNA detection |
| C473 | AGTCACGGGCCCTTACA |  |
| *ACT* | TCTTCCAGCCATCCTTCTTG | For qPCR |
|  | CGGTGATTTCCTTCTGCATT | XM_019042718.1 |
| *COI* | TGATTATTGCCGTTCCTACA | GQ371165.1 |
|  | CATCTACAGAAGAGTTACCAAG |  |
| *OBP1* | AAGTGCTTGACGGATTATTAC |  |
|  | GCATCATATTATCGCAGTGT |  |
| *OBP2* | CTCTTATTGGTCTATTTCTCGTT |  |
|  | CTTCTTCTTCTGGCATTGG |  |
| *OBP3* | CTATCTCGGTTCAGTTCCA |  |
|  | TGTCTTTCCACTCGCTAT |  |
| *OBP4* | CCACGATGTCTTGAGGAT |  |
|  | GCCAAGGAACACTTCATAC |  |
| *OBP5* | AAGTAAAGGCTGTGGATGA |  |
|  | CGAGTAATAGTTGTTGTCTTGA |  |
| *OBP6* | GGAGGAAGGAGTGAATGAC |  |
|  | GTATCTCCACCTGTATTGCTA |  |
| *OBP7* | TCGAATCAGATGCAGAGGGTG |  |
|  | TATCCGGGGGACTCATTCCA |  |
| *OBP8* | TGATGGCGTGTCTTATGA |  |
|  | CTGAGGTTGAGTGCTGTA |  |
| *CSP1* | GTCCTCTCGGCCGATACCTA |  |
|  | AAGCGGTTTTCAAAGCGTCG |  |
| *CSP2* | AGTTCTCGTGGTGTTGTG |  |
|  | GAGTAAGCAGGTGAGGTAG |  |
| *CSP3* | AGTGCGCCAAGTGTACTGAA |  |
|  | ATTTGGCCTCCAATTTCGCC |  |
| *CSP4* | TTCCAGACCTGACCGAAACG |  |
|  | CTCTTGTTGTCCGGGTCGAA |  |
| *CSP5* | AAACGCCTCATCCGCAACTA |  |
|  | CTTGGCGCATTCCGTTTTCA |  |
| *CSP6* | TGTTCGGAGAGCATAGTGCG |  |
|  | GCATGCACTCGACAAGCATC |  |
| *CSP7* | CCGACGGCTTAAAACATT |  |
|  | AGGACCTTCTGGATCTGT |  |
| *CSP9* | CGTGGTTGTTCTGGTCTG |  |
|  | AGGCAGTTGAAGTAGTTATCC |  |
| *CSP10* | ACGCTTTGGAAACAACTTG |  |
|  | GCCGAGGAATGCTTCTTC |  |
| *CSP11* | GTCCTTGCACTAACGAGGGG |  |
|  | AACTGTGCGCACTATCCTCC |  |
| *CSP12* | TTGGTGGAATCAAGGTGCGT |  |
|  | GAGCTCTTGGTATTCCCTCGG |  |
| *CSP13* | CGTGGACCGAGTTCTCAACA |  |
|  | TTCTGCTTCTCCGTGCACTT |  |
| *ETTA2* | CGATTCCAACCAGCAAGA | XM_019045036.1 |
|  | ATCCGAACATTACGCAGAA |  |
| *NHP* | CACCGTTGAGAAGCACAT | BTA004935.1 |
|  | GGAGCATGAGATGAGACAG |  |
| *vGlut* | AACGCCTTCTTCCATTCC | XM_019048530.1 |
|  | GCACGATCACTGTCATCA |  |
| *EAAT1* | CGGAGCCACAATCAACAT | XM_019051271.1 |
|  | TCCAGAAGCCAATCAACAG |  |
| *ATPaseSub1* | AGAATGGACTTACAGAGACAG | BTA022169.1 |
|  | GACAAGAAGCAAGCAAGAG |  |
| *GAT1* | TGCTTCTATTCATCGGTCTC | XM_019054112.1 |
|  | AATCCACTCACTGCGTATG |  |
| *NCh* | GTCAGTCACATCCAGAGG | BTA000397.1 |
|  | GACTCGTCTTCAGGCATT |  |
| *SVAT* | GTGCGGTTGAATAATGTTGA | BTA004529.1 |
|  | AACTACGGTGGTGAGGAG |  |
| *GD2* | TTGCCTCCATCCTTACCA | XM_019056606.1 |
|  | CTACACAATCATCCTCTTCTG |  |
| *GD1* | ATGCCTCCAGATTCCAATG | XM_019055761.1 |
|  | TCAAGCGACCGTAAGAGA |  |
| *GABAS2* | GTGATGGATGTGGTGATGT | XM_019049550.1 |
|  | GCTTGAACGAGAGTGACA |  |
| *NPP* | CGGAAACTTCGCCTACAA | BTA009026.1 |
|  | GTGATGACCTTGACCTCAG |  |
| *VGNaC* | GCAATACATCCGCTACGA | XM_019055445.1 |
|  | GACGCAGAACATCATATCAC |  |
| *ORco* | ACTCACTCTCCTTGCTTATC | XP_018916513.1 |
|  | CCTCTGAACCATCATACCAT |  |
| *Caspase1* | TGTTGGAGACGGTATGGA | BTA009205.1 |
|  | ATGAAGACAGTGCTTAATGC |  |
| *Caspase3b* | CATCACGATCAACGGGACCA | BTA015946.1 |
|  | TGTCGATGTGCTGCTCGAAT |  |
| *NLRL1* | GGACAGCAGTTACAGCATT | BTA019063.1 |
|  | TGAGACAAGAGGAGTGGTT |  |
| *NLRL2* | AGTGCCGAGAACAGTGAT | BTA007439.1 |
|  | GGAGGACAGGAGACATAGG |  |
| *NLRL3* | AATCTGAGAGCATCCAAGTT | BTA019063.1 |
|  | GGCAACATCTAATACCATCAC |  |
| *NLRL4* | ATGAAGGTATCCAAGCACTC | BTA012693.1 |
|  | CGGAACCAACTTACGAACT |  |
| *Spaetzle1&2* | TGTTGGCAGGTTCTCAGT | BTA011933.1 |
|  | CGCAGGACTTGAACATCTT | BTA014026.1 |
| *Spaetzle3* | AGGAATAGAAGTTGAGGAGTG | BTA019895.1 |
|  | CGAACAGGAGCCAGATTG |  |
| *Spaetzle4* | GAGGTCGGCAACTATCCA | BTA026504.1 |
|  | GTGGTCGTCGTCTGAGAT |  |
| *Spaetzle5* | AAGGATTCCAAGGCTATGAC | BTA000088.1 |
|  | GTTCGGCAGTAGTGAAGG |  |
| *Spaetzle6* | AATGGACAAAGGGCGTAG | BTA010665.1 |
|  | CAATGGAAGGACACATAGGA |  |
| *dsGFP* | CTCGTGACCACCCTGACCTAC | dsRNA synthesis |
|  | GTTCACCTTGATGCCGTTCTT |  |
|  | T7-CTCGTGACCACCCTGACCTAC |  |
|  | T7-GTTCACCTTGATGCCGTTCTT |  |
| *dsCaspase1* | TTCGGACAGCCAATTCAG |  |
|  | ACTCTCACCAAGCAATCG |  |
|  | T7-TTCGGACAGCCAATTCAG |  |
|  | T7-ACTCTCACCAAGCAATCG |  |
| *dsCaspase3b* | GATGGAGCGACAGATGAC |  |
|  | GGCGAGCAGAAGTTGAA |  |
|  | T7-GATGGAGCGACAGATGAC |  |
|  | T7-GGCGAGCAGAAGTTGAA |  |
| *dsNLRL4* | GGCATAGGAAGTATTGAAGG |  |
|  | TCAGGAAGTCAGGTAGAGA |  |
|  | T7-GGCATAGGAAGTATTGAAGG |  |
|  | T7-TCAGGAAGTCAGGTAGAGA |  |
| *dsSpaetzle1&2* | CGACGCCTACCCTACTAA |  |
|  | GGATTTCCTTGCCGAAGA |  |
|  | T7-CGACGCCTACCCTACTAA |  |
|  | T7-GGATTTCCTTGCCGAAGA |  |
| V1 probe | Cy5-GGAACATCAGGGCTTCGATA | For FISH |

^a^T7=5’-TAATACGACTCACTATAGG-3’
